# Community participation in mosquito breeding site control: an interdisciplinary mixed methods study in Curaçao

**DOI:** 10.1186/s13071-017-2371-6

**Published:** 2017-09-19

**Authors:** Jelte Elsinga, Henry T. van der Veen, Izzy Gerstenbluth, Johannes G. M. Burgerhof, Arie Dijkstra, Martin P. Grobusch, Adriana Tami, Ajay Bailey

**Affiliations:** 1 Department of Medical Microbiology, University of Groningen, University Medical Center Groningen, Groningen, The Netherlands; 2 Faculty of Spatial Sciences, University of Groningen, Groningen, The Netherlands; 3 Curaçao Biomedical & Health Research Institute, Willemstad, The Netherlands Curaçao; 4 Epidemiology and Research Unit, Medical and Public Health Service of Curaçao, Willemstad, The Netherlands Curaçao; 5 Department of Epidemiology, University of Groningen, University Medical Center Groningen, Groningen, The Netherlands; 6 Department of Social Psychology, University of Groningen, Groningen, The Netherlands; 7 Department of Infectious Diseases, Center of Tropical Medicine and Travel Medicine, Academic Medical Center, University of Amsterdam, Amsterdam, The Netherlands; 8 Population Research Center, Faculty of Spatial Sciences, University of Groningen, Groningen, The Netherlands; 9 Dr. T. M. A. Pai Endowed Chair in Qualitative Methods, Manipal University, Manipal, India

**Keywords:** Mosquito breeding site control, Community mobilization, Curaçao, Chikungunya, Dengue, Mixed methods, Theory of planned behaviour, Health belief model, Integrated vector control, *Aedes aegypti*

## Abstract

**Background:**

As the arboviral diseases dengue, chikungunya and Zika emerge in the Americas, so does the need for sustainable vector control policies. To successfully achieve mosquito control, joint efforts of both communities and governments are essential. This study investigates this important, but by-and-large neglected topic.

**Methods:**

In June and July 2015, a cross-sectional mixed methods study applying a survey questionnaire (response rate of 82.5%; *n* = 339), in-depth interviews (*n* = 20) and focus group discussions (*n* = 7; 50 participants) was performed in Curaçao. The study was designed based on an integrated theoretical framework of the Health Belief Model and the Theory of Planned Behaviour.

**Results:**

Participants showed a good knowledge of, and a high-level performance of mosquito breeding site control (MBSC) practices. Personal protection against mosquitoes (e.g. topical repellents) was perceived as relatively less effective thus practiced to lower extent compared to MBSC practices (i.e. larval source management). A lower intention to perform MBSC was independently associated with: (i) satisfaction on governmental MBSC (*P* = 0.012); (ii) barriers to perform MBSC practices, i.e. ‘Government doesn’t control other breeding sites’ (*P* = 0.005), ‘Don’t know how to control breeding sites’ (*P* = 0.041), and ‘a mosquito does not transmit dengue’ (*P* = 0.016), (i﻿ii) attitudes towards MBSC (*P* = 0.001) and self-efficacy (person’s perceived ability to act) to perform MBSC (*P* = 0.002). Mixed-methods evidence highlights three possible ways of improving community participation in MBSC. First, it highlights the need for ongoing media coverage, targeting (i) communities’ perceptions on transmission routes of dengue and chikungunya, and (ii) presence of car tires in yards. Secondly, it shows that promotion of governmental activities in MBSC can enhance MBSC of communities, if people develop a sense of responsibility to perform MBSC at their own properties. Thirdly, this study describes the presence of key persons in communities, who could be engaged in mosquito control policies to improve MBSC in neighbourhoods.

**Conclusion:**

This study reveals gaps between policy and communities’ lived realities. These gaps might be overcome with the proposed interventions, resulting in a higher performance of MBSC in the community in Curaçao. Furthermore, this study shows how interdisciplinary mixed methods research can provide important, comprehensive, and in-depth insights to inform mosquito control policies.

**Electronic supplementary material:**

The online version of this article (10.1186/s13071-017-2371-6) contains supplementary material, which is available to authorized users.

## Background

The arboviral diseases dengue, chikungunya and Zika impact seriously on public health in endemic countries. Untreated dengue can evolve into severe variants and eventually cause shock or death [1]. Chikungunya is known for its chronic, lingering, mainly musculoskeletal complaints with a major impact on quality of life [2–6]. Zika is ill-famed for congenital Zika syndrome [7], Guillain-Barré syndrome frequency [8], sexual transmissibility [9] and a rare but life-threatening immune-induced thrombocytopenia [10].

These viruses are transmitted by *Aedes* spp., mainly *Ae. aegypti* and *Ae. albopictus*, that flourish in large parts of Southeast Asia, the Americas and Africa, and beyond. In these regions, dengue outbreaks occur on a regular basis [11]. In Curaçao, where dengue is endemic for all four serotypes [12], chikungunya caused a major outbreak in 2014–2015 where approximately 50–75% of the population was infected [6]. High proportions of chikungunya infected individuals (64%) still suffered from the long-term effects of this disease [6]. In January 2016, the first locally transmitted Zika case in Curaçao was reported, heralding an epidemic [13].

Despite the high burden of disease that dengue, chikungunya and Zika cause, marketed vaccines are not available yet. Treatment strategies for all three diseases currently focus on symptom relief. Outdoor personal protection against mosquitoes relies mainly on topical repellents, but these face numerous practical concerns and are often not suitable for efficient long-term use [14, 15]. Meanwhile, the only effective way to reduce the burden of these diseases is prevention, which is mainly dependent on vector control [16, 17].

New methods of vector control using genetic manipulation of *Ae. aegypti* or endosymbiotic bacteria are promising for large-scale field application [18], but are not yet widely used for vector control. Efficacy of long-lasting insecticides applied to curtains or bed nets is poor [19], given the day-biting behaviour of the *Aedes* spp. Application of insecticides is challenged by growing resistance to temephos and pyrethroids of larvae and adult mosquitoes of the *Ae. aegypti* [20]. In the meantime, larval source control remains an effective mosquito control strategy [18, 21].

Identifying and eliminating standing pools of water on a large scale is impractical, expensive and not suitable for sustainable vector control, if conducted solely by government bodies [18]. Meanwhile, examples of mosquito control strategies with community involvement are successful in the short and long term [21–24]. Consequently, complimentary efforts are needed from centralized (governmental) initiatives and the community to enhance effectiveness and sustainability of mosquito control methods [16, 21].

It remains challenging to achieve community participation in mosquito breeding site control (MBSC). Preventive health behaviours are driven by many social and psychological factors, which need to be addressed for forging and implementing of an effective health policy [25, 26]. As crucial as it is, community participation in vector control for dengue and chikungunya remains a neglected topic in scientific literature. The scarcity of community-based, mixed-method approaches using theoretical frameworks in this field are illustrative for the detachment of theory and practice, recognized as a problem in global health research [27–30].

Several health behaviour theories have been developed to understand determinants of behaviour and to provide a basis for effective health policy [25]. Two important theories for preventive behaviour are the Health Belief Model (HBM) and the Theory of Planned Behaviour (TPB). Both theories are validated, and effectively applied in preventive behaviour interventions. Central concepts in the HBM are the ‘perceived susceptibility’ and the ‘perceived severity’ of the condition, leading to the ‘perceived threat’ of the condition. The HBM proposes that the perceived threat of a condition, the perceived benefits and barriers of preventive behaviour, the self-efficacy (a person’s perceived ability to perform the behaviour) and the cues to action predict behaviour [30, 31] (Additional file 1: Figure S1). The Theory of Planned Behaviour (TPB) combines the attitude towards behaviour, subjective norms and perceived behavioural control, resulting in behavioural intentions and behaviour [32]. The HBM and TPB are theories with unique value for health-promotion interventions. Hence, a combination of theories may result in the most effective interventions [25, 33]. In this study, the HBM was applied to understand the risk perceptions of participants and by combining the TPB we were able to measure their perceived control and subjective norms that influence intentions for MBSC.

To our knowledge, no studies combining qualitative and quantitative research methods based on the TPB or HBM to investigate community participation in MBSC have been published to date. We address this knowledge gap here by (i) describing communities’ perceptions and practices of preventive behaviours in MBSC; (ii) analysing communities’ behavioural intentions to control mosquito breeding sites, based on the TPB and the HBM; and (iii) proposing targets for health interventions to enhance community participation in MBSC. The study aims were addressed using qualitative and quantitative approaches on sociological, psychological and epidemiological grounds. This study design enables an in-depth, comprehensive and interdisciplinary understanding of the topic, to date unique in its field.

## Methods

### Study design

In June and July 2015, a cross-sectional mixed method study using individual questionnaires, in-depth interviews (IDIs) and focus group discussions (FGDs) was performed to assess community participation in MBSC in Curaçao. The study was designed based on an integrated theoretical framework of the HBM and TPB (Additional file 1: Figure S1).

### Study site

**Fig. 1:**
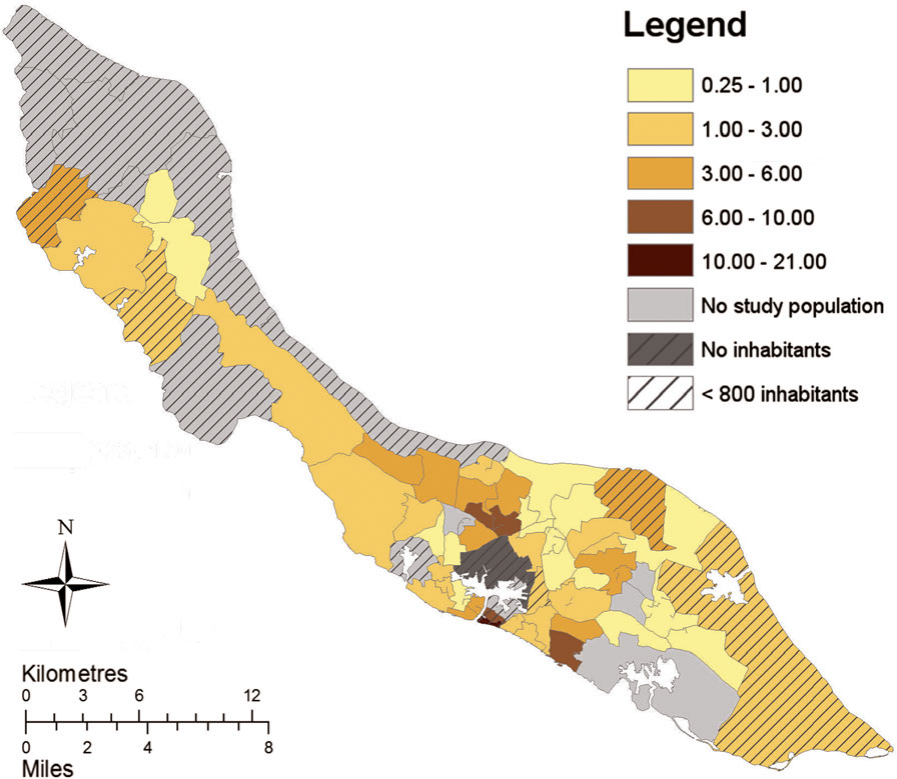
Distribution of the study population among geozones of Curaçao, mean number of cases per 1000 inhabitants. Reproduced and modified from Jelte Elsinga et al. Long-term chikungunya sequelae in Curaçao: burden, determinants and a novel classification tool. *Journal of Infectious Diseases* (2017) jix312, https://doi.org/10.1093/infdis/jix312 [6]. Published by Oxford University Press on behalf of the Infectious Diseases Society of America (IDSA), online at: https://academic.oup.com/jid/article/3926074/Long-term-chikungunya-sequelae-in-Curacao-burden?searchresult=1. This material is covered by a Standard License only. For permissions please email:journals.permissions@oup.com. With permission of Oxford University Press

Curaçao is a Caribbean island in the southern Caribbean Sea located around 100 km off the Venezuelan coast. In October 2010, Curaçao became an autonomous country within the Kingdom of the Netherlands [34]. Curaçao has a surface area of 444 km^2^ and a population of approximately 150,000 inhabitants [35]. It is home to people from different ethnic backgrounds, with an Afro-Caribbean majority [36]. Most of the population resides in the capital Willemstad and its surroundings, located in the central-south part of the island (Fig. 1), the main economic area of the country [35]. Curaçao is a relatively wealthy Caribbean island with a GDP per capita of 22,600 dollar (2012). It has a semi-arid climate with a rainy season from September to January and a dry season from February to June [37, 38]. Generally, all people have access to potable tap water which means that water storage is limited. The central government of Curaçao is responsible for vector control in all neighbourhoods. Vector control activities are performed by the vector control unit of the government. Routine surveillance concern inspection and application of larvicides and fogging at sites at risk for mosquitoes. During epidemics these activities are intensified and focused on properties around suspected arboviral transmission.

### Quantitative methods

#### Study population

Adult subjects with a serologically or clinically confirmed chikungunya infection (of which two were self-diagnosed) during the 2014–2015 chikungunya epidemic were selected from a representative patient sample from 20 general practitioners across the country. Eligible individuals were invited and interviewed at their homes.

#### Data collection

An individual questionnaire containing pre-coded questions was designed in Dutch. After a pilot-study, it was adapted and translated into Papiamentu, Spanish and English. Training was provided to interviewers prior to field mobilization. All interviewers were local, experienced interviewers working for the Central Bureau of Statistics of Curaçao and speaking all four mentioned languages. The questionnaire addressed socio-demographic characteristics and chikungunya chronic disease persistence by applying the Curaçao Long-term Chikungunya Sequelae Score (CLTCS Score), to measure perceived severity of long-term chikungunya disease. This score was calculated using four (5-point Likert-item) questions. More information on the methodology of the scale can be obtained from a recent publication [6].

##### Constructs of the HBM and TPB

The behavioural target was defined as follows; ‘to check the house and yard for mosquito breeding sites every week and eliminate the breeding sites if necessary, in the coming rainy season’. This behavioural target was measured by assessing the Behavioural Intention to perform Mosquito Breeding Site Control (BIMBSC) (Additional file 2). The constructs of the HBM and TPB and their modifying variables (satisfaction on government’s mosquito control action, and knowledge on transmission route of chikungunya and dengue) were measured using multiple five-point Likert items or binary items which were analysed separately or merged into a Likert scale after analysis for internal consistency. Questions were adapted from literature where possible [33, 39].

##### Knowledge

Participants indicated from which sources they received their information relating to chikungunya and dengue among the presented media and education sources. Two ‘interpersonal sources’ were also assessed: general practitioner and family/friends/neighbours. The subject’s perception of chikungunya and dengue transmission routes was tested by asking him/her to indicate all possible transmission routes of these diseases among the presented options.

##### Attitudes and behaviours towards personal protection and mosquito breeding site control

Data on perceptions and performance of personal protection against mosquitoes and MBSC practices was obtained. Participants rated proposed measures on their effectiveness and frequency of performance, applying a five-point Likert item.

#### Data analysis

SPSS Data Entry Station (SPSS Inc. 1996–2003, version 4.0.0) was used for quantitative data entry. Data were checked for consistency and analysed anonymously. Participants were divided by geozone [35] (neighbourhood), which were visualized in Fig. 1 using ArcGIS (ArcGIS Desktop: Release 10.3. Redlands, CA: Environmental Systems Research Institute). Associations between categorical variables were analysed using Chi-square test or Fisher’s exact test when appropriate. Continuous data was compared using a Mann-Whitney U-test or a Student’s t-test. The concepts of the HBM and TPB were tested for their internal consistency using the Cronbach’s Alpha test. If the Cronbach’s Alpha was > 0.60, the items were combined resulting in a Likert scale representing the measure for the corresponding construct of the HBM/TPB. If Cronbach’s Alpha was < 0.60, items were analysed separately, or only the most representative item to measure the concept was used. The questions assigned to constructs of the HBM/TPB are presented in Additional file 2, and their Cronbach’s Alpha in Additional file 1: Table S1. All concepts of the HBM and TPB and the modifying variables were Z-transformed, and correlations were performed using Spearman’s rho. A binary logistic regression was performed to identify which concepts of the HBM and TPB were independently associated with the BIMBSC. Significance was determined at 5% level. Data were analysed using SPSS (SPSS Inc., version 22.0, Chicago, Illinois).

### Qualitative study methods

#### Study population

Participants of the IDIs were adult laboratory-confirmed chikungunya patients. Concerning the FGDs, seven representative population groups (50 participants in total) of Curaçao were selected based on socio-economic status (Additional file 1: Table S2): (i) residents born in the Netherlands; (ii) local youth; (iii) interviewers of the survey; (iv-vii) people from the neighbourhoods of Rooi Santu, Seru Fortuna, Souax and Koraalspecht. The focus group with the survey interviewers aimed at understanding underlying reasons for particular survey results. Participants for the qualitative methods were recruited *via* snowballing (recruitment strategy to recruit participants who are difficult to identify), key informants, and *via* neighbourhood centres.

#### Data collection

Qualitative research methods consisted of IDIs and FGDs based on the Grounded Theory. The Grounded Theory is an analytical strategy to make visible the steps in analyses, to move from data to theoretical explanations [40]. The HBM and the TPB were used to develop a theoretical framework, which is presented in Additional file 1: Figure S1. Interview guides were made based on this framework and adapted after pilot interviews. The FGDs consisted of 4–10 individuals with similar socio-economic backgrounds. The FGDs were applied in Dutch or Papiamentu, depending on participant(s) preferences. Interviews were recorded, translated, transcribed and analysed using codes and code families (see below).

#### Data analysis

Qualitative data were analysed using Atlas.ti (version 7.5.4). Data were examined using codes, which refer to an issue, topic, idea or opinion evident in the data [40]. We employed two cycles of inductive (emerging from data) and deductive (pre-defined from theory) coding. In the first cycle of analysis, 20 codes were used when analysing the FGDs and IDIS. These codes were assigned to 9 code families, which were analysed in the second cycle of analysis. The code families represented perceptions towards: actions of the government in mosquito control, personal protection against mosquitoes, transmission route of chikungunya, information sources on chikungunya/dengue, community initiatives in mosquito control, performance of MBSC, barriers to MBSC, value of MBSC, and waste management.

## Results

### General characteristics of the study population

A total of 411 individuals were invited in June and July 2015 to join this study, of which 339 participated (response rate: 82.5%). Table 1 summarizes the socio-demographic characteristics of the study population. The reasons for non-contacting (selected individuals who were not reached) and non-response were presented elsewhere [6]. Fig. 1 shows the mean number of participants per 1000 inhabitants per geozone. The BIMBSC score ranged from 3 (lowest intention) to 15 (highest intention) (Q1 = 12; median = 15; Q3 = 15). Of the participants, 63.0% (*n* = 208) scored the highest possible BIMBSC score. The characteristics of the participants of the IDIs and FGDs are presented in Additional file 1: Table S2.

**Table 1:** Socio-economic characteristics of the study population, stratified by their score of behavioural intention to perform mosquito control (BIMBSC score)

	Total (*n* = 339)	BIMBSC score < 15 (*n* = 122)	BIMBSC score = 15 (max.) (*n* = 208)	*P*-value^a^
	*n* (%)	*n* (%)	*n* (%)	
Age				
18–40 years	75 (22.1)	32 (26.2)	43 (20.7)	
41–60 years	172 (50.7)	54 (44.3)	111 (53.4)	
> 60 years	92 (27.1)	36 (29.5)	54 (26.0)	0.263
Sex				
Female	247 (72.9)	87 (71.3)	154 (74.0)	
Male	92 (27.1)	35 (28.7)	54 (26.0)	0.590
Education				
Illiterate/primary school	80 (23.6)	25 (20.5)	49 (23.6)	
Secondary school	128 (37.8)	52 (42.6)	75 (36.1)	
Intermediate vocational school	84 (24.8)	30 (24.6)	53 (25.5)	
University (of applied sciences)	47 (13.9)	15 (12.3)	31 (14.9)	0.663
Occupation^b^				
Unemployed/student/housewife/voluntary	63 (18.6)	17 (14.0)	44 (21.2)	
Paid job (domestic or manual)	144 (42.6)	53 (43.8)	87 (41.8)	
Paid job (not domestic nor manual)	67 (19.8)	25 (20.7)	40 (19.2)	
Retired	64 (18.9)	26 (21.5)	37 (17.8)	0.427
Income^c^				
0–999 ANG^d,e^	35 (10.5)	15 (12.8)	20 (9.7)	
1000–2499 ANG	136 (41.0)	50 (42.7)	82 (39.6)	
2500–4999 ANG	118 (35.5)	36 (30.8)	78 (37.7)	
> 5000 ANG	43 (13.0)	16 (13.7)	27 (13.0)	0.592
Disease status chikungunya (CLTCS)				
Recovered	126 (37.2)	44 (36.1)	81 (38.9)	
Mildly affected	121 (35.7)	48 (39.3)	71 (34.1)	
Highly affected	92 (27.1)	30 (24.6)	56 (26.9)	0.635

^a^ *P*-value corresponds to the comparison of the proportions between the groups BIMBSC <15 and BIMBSC = 15 (maximum score)

^b^Total is 338, total < 15 group is 121

^c^Total is 332, total < 15 group is 117, total 15 group is 207

^d^Antillian Guilder; 1 ANG = 0.56 USD

^e^Minimum wages 2015 = 1420 ANG (based on a 40 h workweek). Nine participants missed data on their BIMBSC and were excluded from analysis

### Communities’ attitudes and practices towards MBSC

**Fig. 2:**
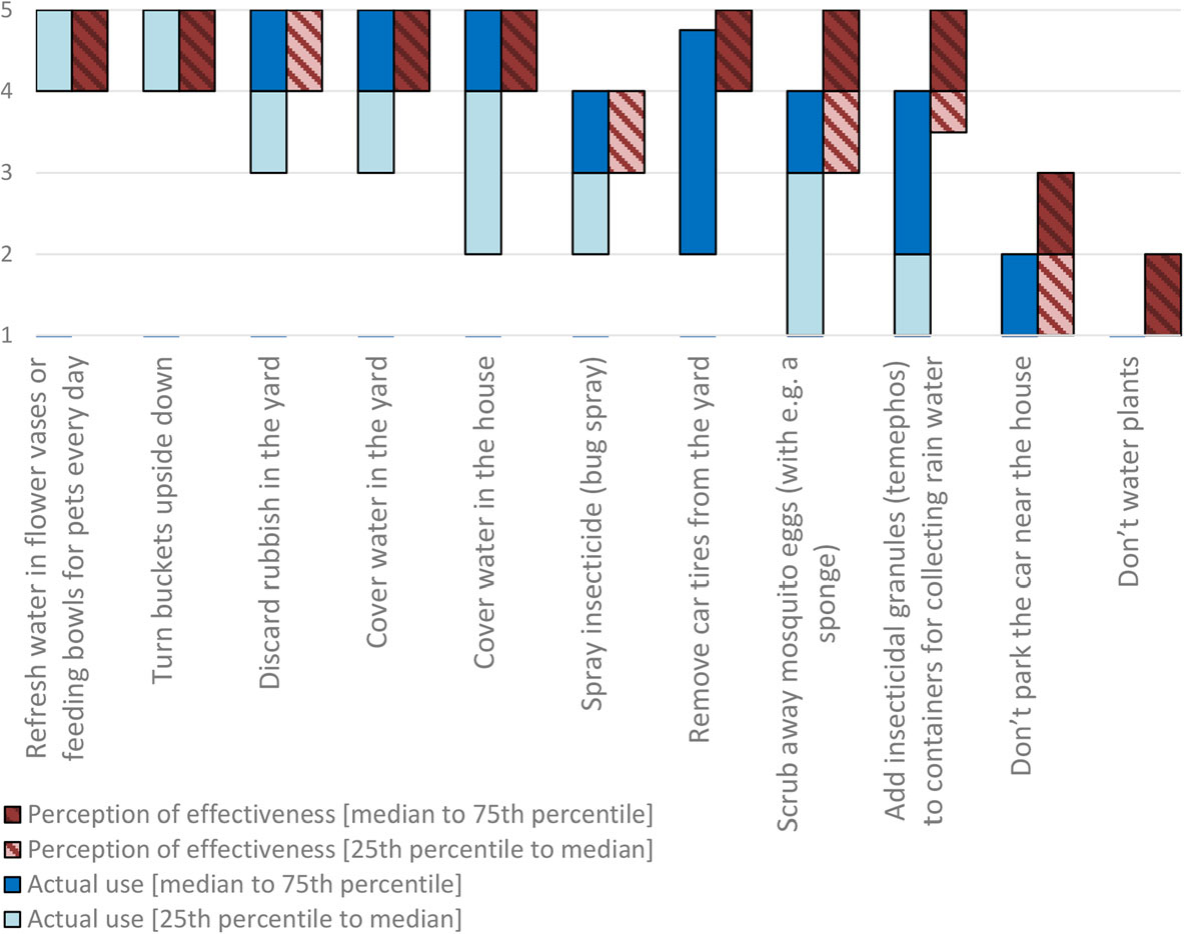
Measures taken by the community preventing mosquitoes from breeding in yards/houses (perceptions of effectiveness and actual use). The blue bar represents the actual taking of a precautionary measure (1, never; 2, sometimes; 3, regularly; 4, often; 5, always), whereas the red bar represents the perceived probability that the measure prevents mosquitoes from breeding (1, not at all; 2, does not; 3, maybe; 4, does; 5, definitely). The bottom edge shows the 25th percentile, the top edge shows the 75th percentile and separation of light and dark (blue or orange) shows the median. When the lighter part is not visible, the median and the 25th percentile coincide in the same value. When the darker part is not visible, the median and the 75th percentile coincide in the same value

**Fig. 3:**
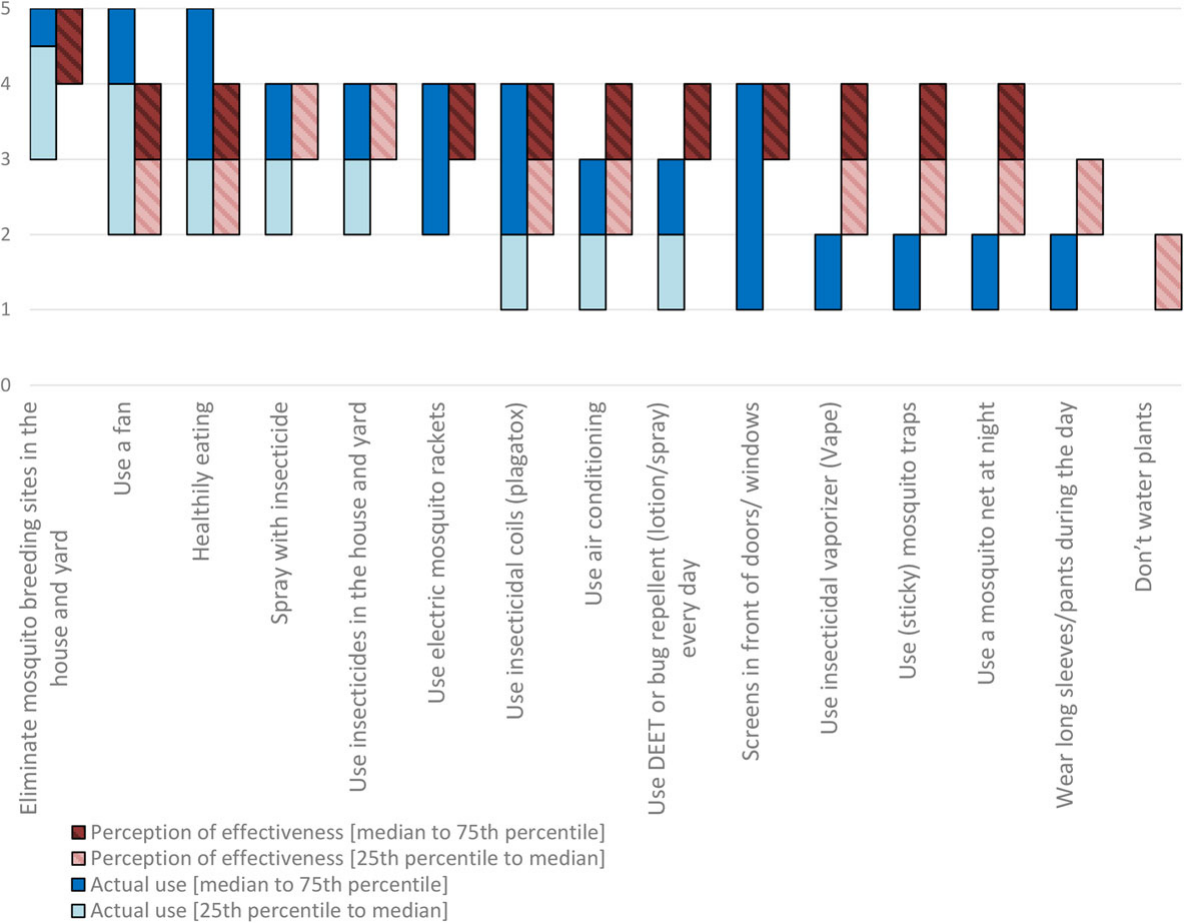
Measures taken by the community preventing oneself from being bitten by mosquitoes (perceptions of effectiveness and actual use). The blue bar represents the actual taking of a precautionary measure (1, never; 2, sometimes; 3, regularly; 4, often; 5, always), whereas the red bar represents the perceived probability that the measure prevents the mosquitoes from biting them (1, not at all; 2, does not; 3, maybe; 4, does; 5, definitely). The bottom edge shows the 25th percentile, the top edge shows the 75th percentile and separation of light and dark (blue or orange) shows the median. When the lighter part is not visible, the median and the 25th percentile coincide in the same value. When darker part is not visible, the median and the 75th percentile coincide in the same value

Different possible measures preventing mosquitoes from breeding and preventing people from being bitten by mosquitoes were assessed for effectiveness and actual use. The answers were ordered by actual use and are presented in Figs. 2, 3; Additional file 1: Tables S3 and S4. Concerning the MBSC measures, people valued the measures preventing stagnant water as most effective. The majority stated that they exercised these measures ‘often’ (score = 4) or ‘always’ (score = 5). Those who possessed car tires indicated that they removed them infrequently (median: 2 ‘sometimes’), while in general this was perceived as a very effective precaution. Spraying insecticides, scrubbing away mosquito eggs and adding Abate [insecticidal granules (temephos)] to water containers were perceived as effective measures, but to a lesser extent. Consequently, they were also performed in a lower degree (Fig. 2, Additional file 1: Table S3).

### Communities’ attitudes and practices towards prevention of mosquito bites

In general, people perceived the measures to prevent themselves from being bitten as less efficient than the measures to prevent mosquitoes from breeding (Figs. 2, 3; Additional file 1: Tables S3 and S4). The measures were ordered based on the actual use, and presented in Fig. 3. The majority of the participants reported using measures to prevent themselves from being bitten at least ‘regularly’ (score ≥ 3) by eliminating mosquito breeding sites, using a fan, healthy eating, spraying with insecticides and by using insecticides inside the house and in the yard (Additional file 1: Table S4). There was no major variation between the perceived effectiveness of measures. The 25th–75th percentile of the effectiveness of all measures (except for ‘MBSC practices’ and ‘don’t water plants’) scored between ‘sometimes’ (score = 2) and ‘often’ (score = 4).

### Qualitative results concerning repellent use and wearing long-sleeved clothes

Participants expressed concerns regarding protecting themselves by wearing long-sleeved clothes. They narrated that mosquitoes could bite through the clothes and it was too warm to wear long-sleeved clothes. Regarding mosquito repellents, participants expressed different opinions. Some mentioned that they used it always. Others stated that they had stopped using repellents, because of the effort it took to buy and use it, while the repellent only protected them for a relatively short time or had not worked at all. Another barrier to use repellents concerned the perception that the repellents could be toxic if used too often.

### HBM and TPB constructs and BIMBSC

The perceived susceptibility of the participant to the acquisition of chikungunya and dengue was moderately low (Q1 = 13, median = 15, Q3 = 19; range of possible scores: 7–35), while the perceived severity of chikungunya and dengue showed moderately high scores (Q1 = 35, median = 40, Q3 = 45; range of possible scores: 10–50). The scores of the other constructs of the HBM and TPB were assessed as ‘moderately high’ or ‘high’. Tables S1 and S5 in Additional file 1 show the scores of the constructs of the HBM and TPB and their Cronbach’s Alpha value.

#### Barriers towards mosquito breeding site control

The scores of the perceived barriers towards MBSC (1: no barrier at all – 5: fully agree *that the issue is a barrier*) for the community are presented in Table S6 in Additional file 1. The assessed barriers were in general not perceived as major issues, except for the barrier: ‘Government doesn’t control other breeding sites’ (Q1 = 2, median = 4, Q3 = 5).

#### Multivariate analysis of the BIMBSC

To assess the associations between the psychological constructs and the BIMBSC, univariate analyses on the general characteristics were performed between those with a BIMBSC score < 15 (lower intention) *vs* 15 (maximum intention) (Table 1). The concepts of the HBM and the TPB were tested with the BIMBSC score using a Mann-Whitney U-test (Additional file 1: Table S1). Consequently, a binary logistic regression was performed including the variables associated at a significance level of *P* ≤ 0.20. Variables were back-wise eliminated until only significant variables were left. The final model is presented in Table 2.

**Table 2:** Final model of factors independently associated with a maximum intention *vs* a lower Behavioural Intention to perform Mosquito Breeding Site Control (BIMBSC)

	Odds Ratio (95% CI)	*P*-value
Barrier: ‘Don’t know how to control breeding sites’	0.77 (0.59–0.99)	0.041
Barrier: ‘Government doesn’t control other breeding sites’	0.67 (0.51–0.89)	0.005
Attitude towards behaviour (performing MBSC)	2.14 (1.56–2.93)	0.001
Self-efficacy^a^	1.54 (1.17–2.04)	0.002
Satisfaction on governmental MBSC	0.71 (0.54–0.93)	0.012
Believing that dengue is transmitted by a mosquito^b^		
No	1	
Yes	2.93 (1.22–7.05)	0.016

^a^Self-efficacy: the belief that a person is capable of performing the health behaviour

^b^Using the normalized value (*z*-value), OR = 1.38, 95% CI: 1.06–1.79), *P* = 0.016

The construct of the TPB ‘attitude towards the behaviour’ (performing MBSC), the self-efficacy (a person’s perceived ability to perform the behaviour) to perform MBSC and the belief that a mosquito transmits dengue were positively related with a maximum BIMBSC score of 15. On the other hand, satisfaction on governmental breeding site control, and two barriers (‘Don’t know how to control breeding sites’ and ‘Government doesn’t control other breeding sites’) had an independent negative relation with the BIMBSC.

### Intervention strategies to enhance community participation in MBSC

To understand community participation in MBSC, the quantitative data on psychological determinants of BIMBSC and the qualitative data from the IDIs and the FGDs were analysed. These analyses were performed to identify intervention strategies that may enhance community participation in MBSC. Based on the latter analyses, three possible intervention methods to improve the BIMBSC were proposed and presented in the next section.

#### Intervention 1: Media and education sources on chikungunya and dengue promote BIMBSC

**Fig. 4:**
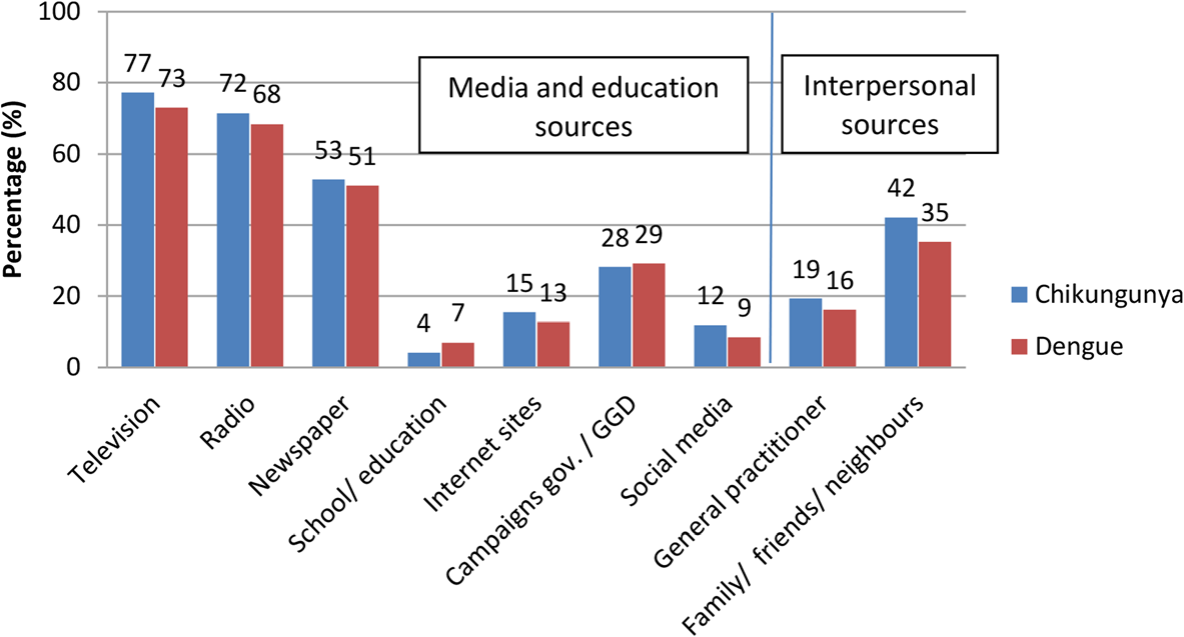
Information sources on chikungunya and dengue. GGD - Medical and Public Health Service of Curaçao

The first proposed intervention is to promote MBSC *via* media and education (cues to action). Most participants referred having heard of chikungunya and dengue through television, radio and the newspaper (Fig. 4 and Additional file 1: Table S7). To explore associations between usage of different media sources and psychological constructs, the reported media and education sources of each individual were added up. These information sources ranged from 0 to 13 sources, with a median of 5 sources (Q1 = 3; Q3 = 8). Separately from the media and education sources, the interpersonal information sources were also assessed (Fig. 4).

**Fig. 5:**
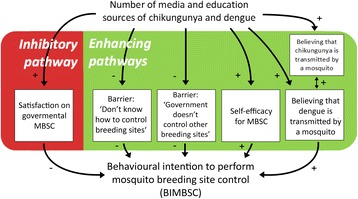
Influence of media and education sources on the BIMBSC *via* psychological constructs. The media and education sources show positive relations with an individual’s BIMBSC *via* the variables in the green box. The media and education sources show a negative relation with an individual’s BIMBSC *via* the variable in the red box. Relations with the BIMBSC are significant independent associations. Relations with the media and education sources are significant associations revealed by a Spearman’s rho test. Knowledge on transmission routes of dengue and chikungunya was significantly correlated (Spearman’s rho = 0.393, *P* < 0.001). A ‘minus’ indicates a negative association, a ‘plus’ indicates a positive association. Self-efficacy refers to the belief that a person is capable of performing the health behaviour

To explore the role of media and education coverage when promoting MBSC, correlations of the number of media and education sources and the determinants of the BIMBSC (Table 2) were analysed using a Spearman’s rho test (Additional file 1: Table S8). The (indirect) associations of media and education coverage on MBSC promotion is presented in Fig. 5.

Media and education promote the BIMBSC in several ways (Fig. 5). First, media and education promoted BIMBSC *via* a positive relation with ‘believing that a mosquito transmits dengue’. The latter positive effect might be stimulated by its positive relation with ‘believing that a mosquito transmits chikungunya’. Furthermore, the BIMBSC was promoted *via* a positive relation with self-efficacy for MBSC, and *via* a direct negative relation with the perceived barriers for MBSC.

Apart from these enhancing effects on the BIMBSC, the amount of media and education sources also showed a negative effect: media and education sources promoted satisfaction on governmental actions on mosquito control, which in turn was associated with a lower BIMBSC.

##### Improving messages to the public: Quantitative analysis of transmission route of chikungunya and dengue

Media and education sources enhanced BIMBSC *via* improvement of perceptions on transmission route of chikungunya and dengue (Fig. 5). In this section, the perceptions on the transmission routes are investigated in depth, with the aim to provide grounds for effective messages to the public.

**Fig. 6:**
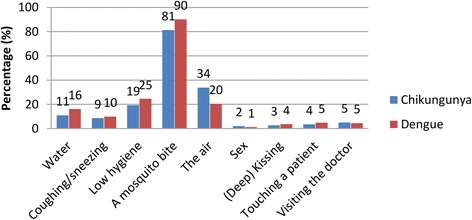
Perceived transmission routes of chikungunya and dengue

Quantitative analysis showed that most people believed that chikungunya and dengue were transmitted by mosquitoes (chikungunya: 81.3% and dengue: 90.1%) (Fig. 6, Additional file 1: Table S9). However, only 49.9 and 54.4% of the participants (referring to chikungunya and dengue, respectively) believed that this was the only route of transmission of both diseases. The remaining participants believed that next to a mosquito, also other transmission routes existed for chikungunya and dengue, of which ‘the air’ (33.8 and 20.4%, respectively), ‘bad hygiene’ (19.3 and 24.6%, respectively) and ‘water’ (11.0 and 16.2%, respectively) were among the most commonly mentioned transmission routes of chikungunya and dengue (Fig. 6, Additional file 1: Table S9).

##### Improving messages to the public: Qualitative analysis of the transmission route of chikungunya

Different opinions about the transmission route of chikungunya were expressed in the group discussions. Among these, the transmission route ‘a virus, *via* a mosquito’ was regularly mentioned. However, doubts about this theory were expressed, based on personal observations. Participants found it difficult to understand that transmission through mosquitoes could have caused such an explosive epidemic, while mosquitoes had been living on Curaçao for many years:

*Man, aged 60-70 years, FGD: ‘because always always always we have mosquitoes here. In the last two years we have chikungunya. That surprises the population. Because always, always we have had mosquitoes here.’*

Hence, different perceptions on transmission routes besides the biomedical one existed, i.e. transmission through the air, (poor) hygiene, a virus (no mosquito), water, or ‘it goes around’ (Additional file 1: Table S10).

#### Intervention 2: Effects of the government’s MBSC actions on individual BIMBSC

##### Quantitative analysis of governmental actions

Two independently associated determinants of the BIMBSC concerned perceptions on governmental actions in mosquito control. Dissatisfaction demonstrated both negative and positive effects on the BIMBSC (Additional file 1: Table S5). The score of the satisfaction on government’s action was moderately low (Q1 = 1, median = 3, Q3 = 4), and demonstrated an independent negative association with the BIMBSC (*P* = 0.012) (Table 2). The lack of government’s action in MBSC was perceived as a barrier (Q1 = 2, median = 4, Q3 = 5), more often for those participants scoring a lower BIMBSC (*P* = 0.005) (Table 2).

##### Qualitative analysis of governmental actions

Most interviews brought up the topic of ‘waste problem’ (garbage) when talking about MBSC. The latter indicates that improper planning regarding waste disposal in Curaçao was linked to the presence of mosquito breeding sites by the community. Accordingly, participants expressed discontent with the government’s role in managing waste in Curaçao, but also regarding the spraying of insecticides and the low visibility of the government’s actions against mosquito breeding sites. Although spraying of insecticides was observed and appreciated by the participants, dissatisfaction existed on the infrequency and unprecise manner of how this spraying was performed.

*Man, aged 70–80 years, FGD: ‘I think that they only have one little trailer with a spray system in Curaçao that drives around the whole island.’ Women, aged 60–70 years, FGD: ‘Yes! With 90 km an hour, I have seen them.’*

Discontent with the government actions led to community mobilization in mosquito control in the neighbourhoods Souax and Seru Fortuna, guided by community key persons.

*Man, aged 60–70 years, FGD: ‘I don’t agree with how he (the minister) talks because he places all the responsibility (to clean their neighbourhoods from waste) on the common people, and sidelines the government completely. (…) Ask for assistance of the neighbourhoods and there are many neighbourhoods willing to organize, there are many people who are willing to seriously put effort in this. And we are willing to help. (…) The last time we fixed the problem ourselves. With a truck and [empty] warehouse* etc. *Without their (the government’s) decision we removed the waste, for nothing. But the government should not think that this is going to be done all the time through us.'*

Other participants argued that MBSC policies of the government would enhance MBSC of the community. Consequently, more communication and exposure of government’s actions against mosquito breeding sites (i.e. surveillance and application of larvicides and adulticides, cleaning of waste) could motivate them to also ‘help’ and to perform MBSC.

Although explanations existed of how discontent with government’s actions in MBSC coincided with community initiatives to control mosquito breeding sites, there was a strong call for more action from the government’s side. Out of the FGDs, a topic list was made on the actions that participants wanted the government to do (Table 3).

**Table 3:** Actions that people want the government to take in mosquito control^a^

Recommended actions	
More spraying of insecticides	
Continuing program of information dissemination through media	
Inform the community on governmental actions on mosquito control	
Active tracing and managing of possible mosquito breeding sites in the neighbourhoods	
Clean up the garbage dumps in the neighbourhoods	
Improve roads	
Educate children in schools on mosquito breeding site management	
An ‘environment-police’, which can be called if illegal waste dump is observed	
More attention to prevention: ‘act proactive, not reactive’	

^a^The order of the topics does not represent importance

#### Intervention 3: Promoting community participation *via* key persons

The participants of the FGDs and IDIs demonstrated willingness to help or cooperate in MBSC, or, maybe even perceived as more important, in cleaning up their neighbourhoods. Different initiatives were described in which communities were mobilized to clean the neighbourhood. Guided by community key persons (which could be individuals or neighbourhood centres), community mobilization was achieved in Souax (described earlier), Rooi Santu, Seru Fortuna and in Piscadera. In Seru Fortuna, a day was organized to clean the neighbourhood. One of the participants stated that this has boosted awareness and willingness to clean houses and gardens in the neighbourhood.

*Man, aged 40–50 years, IDI: ‘We did it. And it was in the news, ‘Oh the people of Seru Fortuna themselves have… eh yes, hand in action and they cleaned their part’, and then the others in their street also did it (cleaned their properties). Ooh it is like that, positive, it reaches others: ‘I am also going to clean my part (property)’. This time I am late (referring to the cleaning of his own property).’*

The participant expressed that more initiatives to clean Seru Fortuna were planned, also targeting the involvement of youth and children. These ‘cleaning days’ would be made as attractive as possible to involve more people by providing food, drinks and a pleasant experience. Another initiative to involve local youth in the cleaning of a street was initiated by an individual. He narrated how he was cleaning one street where a lot of waste is dumped.

*Man, aged 60–70 years, IDI: ‘It is ‘street keep Curaçao clean’. I am cleaning a street where is now a lot of garbage. (…) And these (street) signs I am going to hang up.’*

A school was invited to draw the ‘keep Curaçao clean’ (street) signs in different languages. In this way, local youth was involved in MBSC and awareness of the consequences of poor waste management was raised. The initiatives described above were organized by communities or individuals, independent from coordination of the government.

## Discussion

This study used an interdisciplinary mixed methods approach, to understand perceptions and attitudes of the community towards mosquito breeding site control (MBSC). Furthermore, it aimed to provide a theoretical basis for intervention methods to improve community participation in MBSC, based on the TPB and the HBM. Three intervention methods were proposed to enhance community action towards MBSC: (i) ongoing media attention; (ii) visibility of governmental MBSC policies; and (iii) engagement of key persons in local communities.

Individuals recognized water source management as an effective way to reduce mosquito breeding sites, and stated that they performed this often. This reflects a good knowledge and a high reported performance of MBSC. Participants perceived removing car tires from yards to be highly effective, but relatively few of them reported performing this behaviour. Whilst being difficult to clear from water, car tires provide formidable breeding conditions where mosquitoes flourish [41]. It has been recognized that tires may greatly contribute to a mosquito population [42]. Hence, car tires in yards may represent an important source of mosquitoes on Curaçao. People expressed moderate confidence and reported moderate application of personal protection against mosquito bites. Repellents containing DEET, or wearing long-sleeved clothes are widely recommended in health promotion campaigns for personal protection [43, 44], but were not used often because of inconvenience and doubts on efficacy. Further health messages concerning repellents and long-sleeved clothes might have little value, if the latter two concerns cannot be addressed. Of interest was the widespread belief that ‘healthy eating’ would prevent people from being bitten by mosquitoes. This belief might promote false feelings of safety for those people who eat healthily, and could therefore be targeted in future health interventions.

The behavioural intention to perform mosquito breeding site control (BIMBSC) was high and accordingly, all constructs of the TPB showed high scores. Interestingly, the relatively low perceived susceptibility to dengue and chikungunya did not result in low intentions to perform MBSC. This can be also linked to the fact that the perceived severity of chikungunya and dengue could be one of the motivating factors for performing MBSC. Notwithstanding the high knowledge on, and intention to perform MBSC, it remains vital to improve the preventive behaviours. This is in particular important for these households where little MBSC is performed. The vector (*Aedes* species) can have a relatively short flight range of about 30 m [45]. However, under some circumstances, the flight range can be up to 400 m [46], making one household with mosquito breeding sites a threat for all who live close to this area.

The multivariate analysis revealed the constructs and variables independently associated with the BIMBSC, namely attitudes towards MBSC, self-efficacy of MBSC, believing that a mosquito transmits dengue, satisfaction and dissatisfaction on governmental actions. The strongest independent predictor of the BIMBSC was the attitudes towards MBSC. The presented qualitative data relating to communities’ perceptions and actions provided in-depth insights into these attitudes. Self-efficacy, which is the belief that a person is capable of performing the behaviour, has shown to be in particular important for performing repeated health behaviours [47]. This study shows the same regarding MBSC.

The multivariate analysis, together with the qualitative data were the basis for three possible intervention strategies to improve the BIMBSC: (i) media and education coverage (cues to action); (ii) government’s action; and (iii) use of community key persons.

### Intervention method 1: Education and media coverage (cues to action), grounded in local realities

Education and media coverage warning of epidemics serve as a cue to action for the community to perform preventive activities. The low perceived barriers to perform MBSC and the higher perceived benefits of MBSC and severity of chikungunya and dengue demonstrated in this study, might contribute to increase the favourable impact of cues to action (media and education) [29]. As was suggested in literature [31, 33] (and shown in this study), cues to action enhanced behaviour (BIMBCS) *via* other constructs of the HBM and the TPB (Fig. 5).

Based on our findings, messages to the public should include the transmission route of chikungunya and dengue. Half of the participants believed that the only way of transmitting dengue or chikungunya was *via* a mosquito, while the remaining participants believed that (apart from a mosquito) other transmission routes existed like transmission *via* air, water or through poor hygiene practices. Not recognizing that mosquitoes could transmit dengue had direct negative consequences on BIMBSC. Since knowledge on transmission routes of chikungunya and dengue were correlated, targeting both diseases in campaigns may have a favourable effect on mosquito breeding site control practices. Participants of the FGDs suggested that education in schools can carry messages from children to parents, and might be a suitable intervention to enhance community knowledge of MBSC (Table 3). This is in agreement with other studies [48]. The results of the qualitative analyses revealed different underlying attitudes and beliefs on transmission, which could specifically be targeted in education or in media campaigns (Additional file 1: Table S10).

### Intervention method 2: Governmental action

Cooperation in water source management between community and government is crucial in mosquito control [21]. When people were satisfied with the government’s actions, they showed lower BIMBSC-scores. On the other hand, when they placed the responsibility to control mosquito breeding sites solely on the government, they also had a lower BIMBSC-score (Table 2, Fig. 5). Messages to the public could potentially tackle these phenomena. First, people should be aware that, although crucial, MBSC measures taken by the government are often not enough to adequately control the mosquito population. This means that even if an individual is satisfied with governmental actions, his/her own MBSC remains important. Secondly, the government should be aware that its actions in MBSC have potentially direct and indirect positive effects in mosquito control. The direct effect is achieved *via* the government’s water source management in public spaces. A potential indirect effect may be reached *via* media coverage of the MBSC activities performed by the government, which in turn lowers the barriers for individuals to perform MBSC (Fig. 5). Again, these messages will have higher impact if grounded in local realities [26]. The results of the qualitative research of this study can be used to achieve this (Table 3, Additional file 1: Table S10).

### Intervention method 3: Key persons/community initiatives

The described individual and community initiatives regarding waste management demonstrated the presence of key persons for MBSC in Curaçao. These key persons were locals, willing to act proactively and had the ability to motivate community participation in MBSC. Neighbourhood centres and key persons can play an important role [26, 49] as ‘ambassadors’ of MBSC in their community, by raising awareness and initiating mosquito breeding site control actions. Furthermore, they may provide valuable information to the government concerning community realities with regard to MBSC.

### Limitations and strengths

This study was limited by its cross-sectional design. Future performance of MBSC was predicted using ‘behavioural intentions’. While the behavioural intention is recognized as the best predictor for behaviour, it is no substitute for actual behaviour. Furthermore, the study population of the survey consisted only of people who were (clinically or *via* serology) diagnosed chikungunya patients, which could be considered as a cue to action to perform MBSC. However, nearly all inhabitants of Curaçao had been exposed to this cue to action, by having closely experienced the effects of chikungunya. The study population consisted of more females than males. This might not be a major issue, since women are mainly responsible for housekeeping (and MBSC around the house) in Curaçao. A strength of the study was the interdisciplinary, mixed methods study design. This study design, based on health behaviour theories, is unique in the field of MBSC. Furthermore, a population representing all socio-economic and demographic groups of Curaçao was included in this study. Finally, participants were interviewed in a safe environment, chosen by themselves. The results are considered to be representative for the vast majority of the community of Curaçao.

## Conclusions

The results of this study show how health belief theories serve to understand community participation in MBSC. The outcomes of this study can be used for health policies in Curaçao. The policies should target (i) improving community access to information on transmission routes of dengue and chikungunya; (ii) reducing the practices of storing used car tires in yards; (iii) enhancing visibility of government’s MBSC and the communities’ sense of responsibility to perform MBSC; and (iv) creating a network of local key-persons/ ‘ambassadors’ of MBSC who promote MBSC in their own neighbourhood. The qualitative research provided in-depth understanding of quantitative associations, which helps to target the public in an efficient and culturally sensitive way. To close the gap between science, implementation and communities’ lived reality, it is important that similar mixed-method approaches in different countries are conducted to promote one of the most effective strategies in MBSC, which is community participation.

## Additional files

Additional file 1: Table S1. Univariate analysis of the concepts of the Health Belief Model and Theory of Planned Behaviour *vs* the behavioural intention score to perform mosquito breeding site control: BIMBSC-score (< 15 *vs* 15). **Figure S1.** Theoretical framework Theory of Planned Behaviour and Health Belief Model. **Table S2.** Characteristics of the focus groups. All participants of the in-depth interviews had a laboratory-confirmed chikungunya infection. **Table S3.** Measures to prevent mosquitoes from breeding indoor and/or outdoor. **Table S4.** Measures preventing mosquito bites. **Table S5.** Scores of concepts of the Health Belief Model and Theory of Planned Behaviour. **Table S6.** Barriers for eliminating breeding sites. **Table S7.** Information sources of chikungunya and dengue. More answers were possible. **Table S8.** Spearman’s correlation matrix of concepts significantly associated with ‘number of media and education sources’. **Table S9.** Knowledge on chikungunya and dengue transmission routes. More answers were possible. **Table S10.** Reported modes of infection in focus groups. (DOCX 97 kb)

Additional file 2: Survey instrument Theory of Planned Behaviour and Health Belief Model, ordered by concept. (DOCX 57 kb)

## Abbreviations

BIMBSC: Behavioural intention to perform mosquito breeding site control
CLTCS: Curaçao long-term chikungunya sequelae
FGD: Focus group discussion
HBM: Health Belief Model
IDI: In-depth interview
MBSC: Mosquito breeding site control
TPB: Theory of planned behaviour
