# Supplementary material for: Community participation in mosquito breeding site control: an interdisciplinary mixed methods study in Curaçao
Source: Parasit Vectors. 2017 Sep 19;10:434. doi: 10.1186/s13071-017-2371-6 (PMC5606078; doi:10.1186/s13071-017-2371-6)
Supplement: Supplementary file 1 — Univariate analysis of the concepts of the Health Belief Model and Theory of Planned Behaviour vs the behavioural intention score to perform mosquito breeding site control: BIMBSC-score (< 15 vs 15). Figure S1. Theoretical framework Theory of Planned Behaviour and Health Belief Model. Table S2. Characteristics of the focus groups. All participants of the in-depth interviews had a laboratory-confirmed chikungunya infection. Table S3. Measures to prevent mosquitoes from breeding indoor and/or outdoor. Table S4. Measures preventing mosquito bites. Table S5. Scores of concepts of the Health Belief Model and Theory of Planned Behaviour. Table S6. Barriers for eliminating breeding sites. Table S7. Information sources of chikungunya and dengue. More answers were possible. Table S8. Spearman’s correlation matrix of concepts significantly associated with ‘number of media and education sources’. Table S9. Knowledge on chikungunya and dengue transmission routes. More answers were possible. Table S10. Reported modes of infection in focus groups. (DOCX 97 kb) [file 13071_2017_2371_MOESM1_ESM.docx]

**Additional file 1**

**Additional file 1: Table S1. Univariate analysis of the concepts of the Health Belief Model and Theory of Planned Behaviour vs. the Behavioural Intention Score to perform Mosquito Breeding Site Control (BIMBSC): BIMBSC-score (<15 vs. 15)**

|  | | # of items | Range of score | Cronbach’s Alpha | p-value^a^ | Association^b^ |
| --- | --- | --- | --- | --- | --- | --- |
| **Behavioural intention to perform mosquito breeding site control (BIMBSC)** | | 3 | 1 - 5 | 0.774 | n/a | n/a |
| **Health Belief Model** | | |  |  |  |  |
| Perceived threat | Susceptibility (chikungunya and dengue) | 7 | 1 – 5 | 0.669 | 0.320 | - |
|  | Severity (chikungunya and dengue) | 10 | 1 – 5 | 0.814 | 0.074 | + |
|  | *Cues to action* |  |  |  |  |  |
|  | # of media sources (chikungunya and dengue) | 2 | 0 – 7 | 0.807 | 0.213 | + |
|  | # of interpersonal sources (chikungunya and dengue) | 2 | 0 – 2 | 0.628 | 0.446 | - |
| Perceived benefits^c^ |  | 6 | 1 – 5 | 0.956 | 0.836 | + |
| Perceived barriers | Don’t know how to control breeding sites | 1 | 1 – 5 | n/a | **0.000** | - |
|  | Don’t like to control breeding sites | 1 | 1 – 5 | n/a | **0.012** | - |
|  | Neighbours don’t control breeding sites either | 1 | 1 – 5 | n/a | **0.035** | - |
|  | No mosquitoes at my property | 1 | 1 – 5 | n/a | 0.379 | - |
|  | Government doesn’t control other breeding sites | 1 | 1 – 5 | n/a | 0.051 | - |
|  | No other possibilities for storing waste than in my garden | 1 | 1 – 5 | n/a | 0.102 | - |
|  | More mosquitoes are at nearby mondi’s (forest, country side) | 1 | 1 – 5 | n/a | 0.486 | - |
|  | Someone else controls breeding sites (for me) | 1 | 1 – 5 | n/a | 0.996 | = |
|  | No physical ability | 1 | 1 – 5 | n/a | **0.005** | - |
|  | I am only at home when it’s dark | 1 | 1 – 5 | n/a | 0.308 | - |
| Self-efficacy^d^ |  | 2 | 1 – 5 | 0.873 | **0.000** | + |
| **Theory of planned behaviour** | | |  |  |  |  |
| Attitude towards behaviour | | 6 | 1 – 5 | 0.632 | **0.000** | + |
| Subjective norms | | 9 | 1 – 5 | 0.771 | **0.001** | + |
| Perceived behavioural controls | | 1 | 1 – 5 | n/a | **0.001** | + |
| *Modifying factors* | | | | |  |  |
| Knowledge | Believing that chikungunya is transmitted by a mosquito | 1 | 0 – 1 | n/a | 0.954 | + |
|  | Believing that dengue is transmitted by a mosquito | 1 | 0 – 1 | n/a | **0.002** | + |
| Satisfaction on governmental mosquito breeding site control | | 1 | 0 – 5 | n/a | 0.116 | - |

*^a^The p-value corresponds to the comparison between the maximum vs. a lower BIMBSC;* ^b^*The correlation of the BIMBSC and the tested concept was positive (+), negative (-) or neutral (=); ^c^Perceived benefits – probability that MBSC would prevent the participant, family and neighbours from becoming infected by dengue and chikungunya; ^d^Self-efficacy - the belief that a person is capable of performing the health behaviour*

**Modifying factors**

Perceived susceptibility to chikungunya & dengue

*Demographic variables* (age, sex, education, occupation, income)

*Structural variables*

- Knowledge about transmission routes of chikungunya and dengue - - Knowledge about eradication of breeding sites.

- Satisfaction on government’s actions.

Perceived seriousness (severity) of chikungunya & dengue

*Cues to Action*

- Presentation disease symptoms

- Mass media coverage of epidemics
- Nearby chikungunya / dengue cases

**Subjective norms & self-efficacy**

Self-efficacy

**Attitude towards behaviour**

**Perceived threat**

Behaviour (mosquito breeding site control)

Perceive benefits of mosquito breeding site control

Likelihood of performing mosquito breeding site control

Perceived barriers to mosquito breeding site control

Subjective norms

Perceived behavioural control

**Additional file 1: Figure S1. Theoretical framework Theory of Planned Behaviour and Health Belief Model**

Constructs of the Health Belief Model: Perceived susceptibility – the perception of the risk to acquire the disease/condition; Perceived severity – the perceived seriousness of the disease/condition; Perceived benefits – the perceived positive consequences of performing the health behaviour; Perceived barriers – the perceived obstacles to perform the health behaviour; Self-efficacy – the belief that a person is capable of performing the health behaviour; Cues to action – triggers to engage people in performing the health behaviour.
Constructs of the Theory of Planned Behaviour: Attitude towards behaviour – the evaluation of accessible positive and negative beliefs about the health behaviour; Subjective norms – the perceptions of an individual’s belief about the health behaviour, influenced by significant others; Perceived behavioural control – an individual’s evaluation of the ease or difficulty to perform the health behaviour.**Additional file 1: Table S2. Characteristics of the focus groups**

| Focus group | # participants | # female | # self-reported (former) chikungunya patients | Age range | Socio-economic status of represented neighbourhood/group^a^ |
| --- | --- | --- | --- | --- | --- |
| Residents from the Netherlands | 8 | 6 | 6 | 61-71 | high |
| Local youth | 4 | 2 | 0 | 19-24 | middle |
| Interviewers of the survey | 4 | 3 | 0 | 64-67 | middle-high |
| Rooi santu | 8 | 4 | 7 | 51-80 | middle-high |
| Seru Fortuna | 9 | 8 | 8 | 18-70 | low |
| Souax | 7 | 4 | 1 | 34-72 | low |
| Koraalspecht | 10 | 10 | 9 | 55-97 | low-middle |
| In-depth interviews | 20 | 12 | 20^b^ | 36-87 | low-middle-high |

*^a^Based on local classification (homes, amenities, public services) socio-economic status was arrived at as relevant to the local context in Curaçao.  ^b^All participants of the in-depth interviews had a laboratory-confirmed chikungunya infection.*

**Additional file 1: Table S3. Measures to prevent mosquitoes from breeding indoor and/or outdoor**

|  | **Perception of effectiveness** | | **Actual use** | |
| --- | --- | --- | --- | --- |
|  | *Mean (sd) n* | *Quartiles*  *25 – 50 – 75* | *Mean (sd) n* | *Quartiles*  *25 – 50 – 75* |
| Refresh water in flower vases or feeding bowls for pets every day | 4.21 (0.76) 338 | 4 – 4 – 5 | 4.34 (1.01) 179 | 4 – 5 – 5 |
| Turn buckets upside down | 4.32 (0.79) 338 | 4 – 4 – 5 | 4.23 (1.14) 283 | 4 – 5 – 5 |
| Discard rubbish in the yard | 4.46 (0.64) 338 | 4 – 5 – 5 | 4.13 (0.99) 326 | 3 – 4 – 5 |
| Cover water in the yard | 4.28 (0.78) 338 | 4 – 4 – 5 | 3.65 (1.55) 324 | 3 – 4 – 5 |
| Cover water in the house | 4.06 (0.87) 338 | 4 – 4 – 5 | 3.61 (1.51) 337 | 2 – 4 – 5 |
| Spray insecticide (bug spray) | 3.62 (0.96) 338 | 3 – 4 – 4 | 3.09 (1.25) 338 | 2 – 3 – 4 |
| Remove car tires from the yard | 4.36 (0.77) 338 | 4 – 4 – 5 | 2.93 (1.49) 44 | 2 – 2 – 4.75 |
| Scrub away mosquito eggs (with e.g. a sponge) | 3.82 (1.00) 337 | 3 – 4 – 5 | 2.87 (1.54) 337 | 1 – 3 – 4 |
| Add Abate* to containers for collecting rain water | 3.92 (0.96) 337 | 3.5 – 4 – 5 | 2.68 (1.56) 122 | 1 – 2 – 4 |
| Don’t park the car near the house | 2.25 (1.00) 338 | 1 – 2 – 3 | 1.71 (1.32) 253 | 1 – 1 – 2 |
| Don’t water plants | 1.58 (0.72) 338 | 1 – 1 – 2 | 1.19 (0.61) 295 | 1 – 1 – 1 |

*The score on ‘perception of effectiveness’ reflects the perceived probability that the measure prevents mosquitoes from breeding (1=not at all; 2=does not; 3=maybe; 4=does; 5=definitely). The score of the actual use represents the actual use of the measure (1=never; 2=sometimes; 3=regularly; 4=often; 5=always). *Abacte - insecticidal granules (temephos)*

|  | Perception of effectiveness | | Actual use | |
| --- | --- | --- | --- | --- |
|  | *Mean (sd)* *n* | *Quartiles*  *25 – 50 – 75* | *Mean (sd) n* | *Quartiles*  *25 – 50 – 75* |
| Eliminate mosquito breeding sites in the house and yard | 4.28 (0.82) 337 | 4 – 4 – 5 | 4.07 (1.14) 336 | 3 – 4.5 – 5 |
| Use a fan | 3.16 (1.00) 337 | 2 – 3 – 4 | 3.43 (1.31) 338 | 2 – 4 – 5 |
| Eat healthily | 2.71 (1.24) 336 | 2 – 3 – 4 | 3.07 (1.52) 337 | 2 – 3 – 5 |
| Spray with insecticide | 3.53 (0.95) 337 | 3 – 4 – 4 | 3.15 (1.26) 338 | 2 – 3 – 4 |
| Use insecticides in the house and yard | 3.53 (0.97) 337 | 3 – 4 – 4 | 2.72 (1.25) 337 | 2 – 3 – 4 |
| Use electric mosquito rackets | 3.34 (0.99) 337 | 3 – 3 – 4 | 2.70 (1.34) 338 | 2 – 2 – 4 |
| Use Coils (plagatox) | 3.22 (1.05) 336 | 2 – 3 – 4 | 2.35 (1.34) 337 | 1 – 2 – 4 |
| Screens in front of doors/ windows | 3.26 (0.98) 337 | 3 – 3 – 4 | 2.30 (1.54) 337 | 1 – 1 – 4 |
| Use air conditioning | 2.85 (1.13) 337 | 2 – 3 – 4 | 2.24 (1.44) 337 | 1 – 2 – 3 |
| Use DEET or bug repellent (lotion/spray) every day | 3.38 (1.00) 337 | 3 – 3 – 4 | 2.21 (1.21) 337 | 1 – 2 – 3 |
| Use Vape – insecticidal vaporizer | 3.06 (0.97) 335 | 2 – 3 – 4 | 1.84 (1.17) 337 | 1 – 1 – 2 |
| Use (sticky) mosquito traps | 2.77 (1.07) 335 | 2 – 3 – 4 | 1.81 (1.20) 334 | 1 – 1 – 2 |
| Wear long sleeves/pants during the day | 2.63 (0.98) 336 | 2 – 3 – 3 | 1.77 (1.03) 336 | 1 – 1 – 2 |
| Use a mosquito net at night | 3.18 (1.03) 337 | 2 – 3 – 4 | 1.48 (0.98) 337 | 1 – 1 – 2 |
| Don’t water plants | 1.68 (0.79) 336 | 1 – 2 – 2 | 1.20 (0.64) 338 | 1 – 1 – 1 |

**Additional file 1: Table S4. Measures preventing mosquito bites**

*The score on ‘perception of effectiveness’ reflects the perceived probability that the measure prevents from mosquito bites (1=not at all; 2=does not; 3=maybe; 4=does; 5=definitely). The score of the actual use represents the actual use of the measure (1=never; 2=sometimes; 3=regularly; 4=often; 5=always).*

**Additional file 1: Table S5. Scores of concepts of the Health Belief Model and Theory of Planned Behaviour**

|  | | | *Range of score* | *Mean (sd)* *n* | *Quartiles*  *25 – 50 – 75* |
| --- | --- | --- | --- | --- | --- |
| **Behavioural intention to perform mosquito breeding site control (BIMBSC)** | | | 3-15 | 13.45 (2.58) 330 | 12 – 15 – 15 |
| **Health Belief Model** | | | |  |  |
| Perceived threat | | Susceptibility (chikungunya and dengue) | 7-35 | 16.16 (5.06) 332 | 13 – 15 – 19 |
|  | | Severity (chikungunya and dengue) | 10-50 | 39.69 (6.99) 331 | 35 – 40 – 45 |
|  | | *Cues to action* |  |  |  |
|  | | # of media sources (chikungunya and dengue) | 0-14 | 5.11 (3.01) 328 | 3 – 5 – 8 |
|  | | # of ‘peer’ sources (chikungunya and dengue) | 0-4 | 1.13 (1.12) 328 | 0 – 1 – 2 |
| Perceived benefits^a^ | |  | 6-30 | 21.67 (6.25) 333 | 18 – 20 – 30 |
| Perceived barriers | | Don’t know how to control breeding sites | 1-5 | 1.41 (0.94) 333 | 1 – 1 – 1 |
|  | | Don’t like to control breeding sites | 1-5 | 1.38 (0.90) 333 | 1 – 1 – 1 |
|  | | Neighbours don’t control breeding sites either | 1-5 | 1.35 (0.83) 333 | 1 – 1 – 1 |
|  | | No mosquitoes at my property | 1-5 | 1.74 (1.10) 332 | 1 – 1 – 3 |
|  | | Government doesn’t control other breeding sites | 1-5 | 3.45 (1.61) 333 | 2 – 4 – 5 |
|  | | No other possibilities for storing waste than in my garden | 1-5 | 1.34 (0.85) 333 | 1 – 1 – 1 |
|  | | More mosquitoes are at nearby mondi’s (forest, country side) | 1-5 | 2.10 (1.51) 332 | 1 – 1 – 3 |
|  | | Someone else controls breeding sites (for me) | 1-5 | 2.19 (1.46) 333 | 1 – 1 – 3 |
|  | | No physical ability | 1-5 | 1.65 (1.13) 333 | 1 – 1 – 2 |
|  | | I am only at home when it’s dark | 1-5 | 1.31 (0.79) 333 | 1 – 1 – 1 |
| Self-efficacy^b^ | |  | 2-10 | 8.73 (1.78) 332 | 8 – 10 – 10 |
| **Theory of planned behaviour** | | | |  |  |
| Attitude towards behaviour | | | 6-30 | 27.12 (2.77) 332 | 26 – 28 – 30 |
| Subjective norms | | | 9-45 | 34.65 (5.39) 333 | 31 – 35 – 39 |
| Perceived behavioural controls | | | 1-5 | 3.96 (1.37) 333 | 3 – 5 – 5 |
| *Modifying factors* | | | | | |
| Knowledge | Believing that chikungunya is transmitted by a mosquito | | 0-1 | 0.81 (0.39) 337 | 1 – 1 – 1 |
|  | Believing that dengue is transmitted by a mosquito | | 0-1 | 0.90 (0.30) 333 | 1 – 1 – 1 |
| Satisfaction on governmental mosquito breeding site control | | | 0-5 | 2.70 (1.45) 333 | 1 – 3 – 4 |

*^a^Perceived benefits – probability that MBSC would prevent the participant, family and neighbours from becoming infected by dengue and chikungunya; ^b^Self-efficacy -* *the belief that a person is capable of performing the health behaviour*

**Additional file 1: Table S6. Barriers for eliminating breeding sites**

|  | *Mean (sd)* *n* | *Quartiles*  *25 – 50 – 75* |
| --- | --- | --- |
| Don’t know how to control breeding sites | 1.41 (0.94) 333 | 1 – 1 – 1 |
| Don’t like to control breeding sites | 1.38 (0.90) 333 | 1 – 1 – 1 |
| Neighbours don’t control breeding sites either | 1.35 (0.83) 333 | 1 – 1 – 1 |
| No mosquitoes at my property | 1.74 (1.10) 332 | 1 – 1 – 3 |
| Government doesn’t control other breeding sites | 3.45 (1.61) 333 | 2 – 4 – 5 |
| No other possibilities for storing waste than in my garden | 1.34 (0.85) 333 | 1 – 1 – 1 |
| More mosquitoes are at nearby mondi’s (forest, country side) | 2.10 (1.51) 332 | 1 – 1 – 3 |
| Someone else controls breeding sites (for me) | 2.19 (1.46) 333 | 1 – 1 – 3 |
| No physical ability | 1.65 (1.13) 333 | 1 – 1 – 2 |
| I am only at home when it’s dark | 1.31 (0.79) 333 | 1 – 1 – 1 |

*Barriers were assessed with a 5-point Likert item (1: no barrier at all – 5: fully agree that the
issue is a barrier).*

**Additional file 1: Table S7. Information sources of chikungunya and dengue**

|  | Chikungunya (n=337) | | Dengue (n=329) | |
| --- | --- | --- | --- | --- |
| **Information sources**  chikungunya n=337; dengue n=329 | n | (%) | n | (%) |
| *Media sources* |  |  |  |  |
| Television | 260 | (77.2) | 240 | (72.9) |
| Radio | 241 | (71.5) | 225 | (68.4) |
| Newspaper | 178 | (52.8) | 168 | (51.1) |
| School/ education | 14 | (4.2) | 23 | (7.0) |
| Internet sites | 52 | (15.4) | 42 | (12.8) |
| Government campaigns/ GGD* | 95 | (28.2) | 96 | (29.2) |
| Social media (Twitter/Facebook etc.) | 40 | (11.9) | 28 | (8.5) |
| *Peer sources* |  |  |  |  |
| From the general practitioner | 65 | (19.3) | 53 | (16.1) |
| Family/ friends/ neighbours | 142 | (42.1) | 116 | (35.3) |

*More answers were possible. *GGD= Medical and Public Health
Service of Curaçao*

**Additional file 1: Table S8. Spearman’s correlation matrix of concepts
significantly associated with ‘number of media and education
sources’.**

|  | | Number of media and education sources |
| --- | --- | --- |
| Barrier 1 | Correlation Coefficient | -0.128^*^ |
|  | p-value | **0.021** |
|  | n | 324 |
| Barrier 2 | Correlation Coefficient | -0.130^*^ |
|  | p-value | **0.019** |
|  | n | 324 |
| Self-efficacy^a^ | Correlation Coefficient | 0.127^*^ |
|  | p-value | **0.023** |
|  | n | 323 |
| Stating that a mosquito transmits chikungunya | Correlation Coefficient | 0.191^**^ |
|  | p-value | **0.001** |
|  | n | 328 |
| Stating that a mosquito transmits dengue | Correlation Coefficient | 0.133^*^ |
|  | p-value | **0.016** |
|  | n | 328 |
| Satisfation on government’s mosquito breeding site control | Correlation Coefficient | 0.123^*^ |
|  | p-value | **0.027** |
|  | n | 324 |

**Correlation is significant at the 0.05 level (2-tailed); **Correlation is
significant at the 0.01 level (2-tailed); Barrier 1 = Don’t know how to
control breeding sites; Barrier 2= Government doesn’t control other
breeding sites; ^a^Self-efficacy -* *the belief that a person is capable of
performing the health behaviour***Additional file 1: Table S9. Knowledge on chikungunya and dengue
transmission routes**

|  | Chikungunya (n=337) | | Dengue (n=333) | |
| --- | --- | --- | --- | --- |
| **Transmission routes** chikungunya n=337; dengue n=333 | n | (%) | n | (%) |
| Water | 37 | (11.0) | 54 | (16.2) |
| Coughing/sneezing | 29 | (8.6) | 33 | (9.9) |
| Low hygiene | 65 | (19.3) | 82 | (24.6) |
| A mosquito bite | 274 | (81.3) | 300 | (90.1) |
| The air ‘airu’ | 114 | (33.8) | 68 | (20.4) |
| Sex | 7 | (2.1) | 4 | (1.2) |
| (Deep) Kissing | 9 | (2.7) | 12 | (3.6) |
| Touching a (chikungunya/ dengue) patient | 12 | (3.6) | 16 | (4.8) |
| Visiting the general practitioners’ office | 17 | (5.0) | 15 | (4.5) |

*More answers were possible.*

**Additional file 1: Table S10. Reported modes of infection in focus groups**

| **The air / contagious** | *- Man, aged 20-30 years, FGD: ‘(…) I think, well. Many people have got it. I don’t think that mosquitoes bite so many people. I think that that thing [is] in the air itself so, and that I think.’ - Woman, aged 60-70 years, FGD: ‘It is like when we catch a cold. The air transfers it to other people. (…) someone gets it and the other who lives with him (or her) also gets it (…)’* |
| --- | --- |
| **Hygiene** | *Woman, aged 60-70 years, FGD: ‘It is not the mosquitoes. It is our own hygiene. We are the caregivers of ourselves [we need to take care of ourselves].’* |
| **It goes around** | *Woman, aged 60-70 years, FGD: ‘I once thought, it is like something that goes around. I feel it in my hand and my knee today and my neighbour a bit further away felt it also in his hand and knee. This is how it goes around. I got dengue and it is not comparable.’* |
| **A virus (no mosquito)** | *- Man, aged 40-50 years, FGD: ‘ (…) If it is a thing… let me say we had already mosquitoes and they fly, every day, every moment. A virus it is, it is a snapshot, do you get it? A virus comes and then it goes. But a virus stays in the air and you never know when it will attack you. (…)’ - Woman, aged 60-70 years, FGD: ‘he (chikungunya) gets us in the leg first. It’s a virus. (…) Woman (expressing her doubts): ‘but that a mosquito can bite everyone in the leg…’* |
| **Water** | *Woman, aged 70-80 years, FGD: ‘(…) Just we had the impression that even the water that we drink, or [use to] to wash, can’t that bring the virus more easily to people?’* |
