# Supplementary material for: Community participation in mosquito breeding site control: an interdisciplinary mixed methods study in Curaçao
Source: Parasit Vectors. 2017 Sep 19;10:434. doi: 10.1186/s13071-017-2371-6 (PMC5606078; doi:10.1186/s13071-017-2371-6)
Supplement: Supplementary file 2 — Survey instrument Theory of Planned Behaviour and Health Belief Model, ordered by concept. (DOCX 57 kb) [file 13071_2017_2371_MOESM2_ESM.docx]

| **Behavioural intention to perform mosquito breeding site control (BIMBSC)**  The BIMBSC-score is obtained by adding up the values of the three answers. | | | | | | |
| --- | --- | --- | --- | --- | --- | --- |
| The coming rainy season, I will check my house and yard for mosquito breeding sites every week and eliminate them if necessary… | | | | | | |
|  | 1 | 2 | 3 | 4 | 5 |  |
| I will definitely not do | 🗆 | 🗆 | 🗆 | 🗆 | 🗆 | I will definitely do |
| I will not even try | 🗆 | 🗆 | 🗆 | 🗆 | 🗆 | I will definitely try |
| I’m not planning to | 🗆 | 🗆 | 🗆 | 🗆 | 🗆 | I am planning to |

**Survey instrument Theory of Planned Behaviour and Health Belief Model, ordered by concept
(cues to action and knowledge are described in ‘Methods’)***The titles of the survey instruments were different from the titles presented here.*

| **Susceptibility (chikungunya and dengue)**  Fully agree  Do not agree at all | | | | | |
| --- | --- | --- | --- | --- | --- |
| Check to what extent you agree with the following statements: |  |  |  |  |  |
|  | **1** | **2** | **3** | **4** | **5** |
| I expected to get chikungunya ………………………….................................................. | 🗆 | 🗆 | 🗆 | 🗆 | 🗆 |
| I think I had a greater chance of getting chikungunya compared to others………. | 🗆 | 🗆 | 🗆 | 🗆 | 🗆 |
| Everyone in Curaçao runs a great risk of getting chikungunya ………………………… | 🗆 | 🗆 | 🗆 | 🗆 | 🗆 |
| I think I run the risk of getting chikungunya once more……………………………………. | 🗆 | 🗆 | 🗆 | 🗆 | 🗆 |
| I think I have a greater chance of getting dengue compared to others…………….. | 🗆 | 🗆 | 🗆 | 🗆 | 🗆 |
| Everyone in Curaçao runs a great risk of getting dengue ………………………………… | 🗆 | 🗆 | 🗆 | 🗆 | 🗆 |
| I think I run the risk of getting dengue (once more) ………………………………………… | 🗆 | 🗆 | 🗆 | 🗆 | 🗆 |

| **Severity (chikungunya and dengue)**  Fully agree  Do not agree at all | | | | | |
| --- | --- | --- | --- | --- | --- |
| Check to what extent you agree with the following statements: |  |  |  |  |  |
|  | **1** | **2** | **3** | **4** | **5** |
| Chikungunya is a serious disease……………………………………………………………………… | 🗆 | 🗆 | 🗆 | 🗆 | 🗆 |
| People who get chikungunya can die of it……………………………………………………….. | 🗆 | 🗆 | 🗆 | 🗆 | 🗆 |
| People who have chikungunya are limited in their social activities…………………… | 🗆 | 🗆 | 🗆 | 🗆 | 🗆 |
| An infection with chikungunya leads to loss of income…………………………………….. | 🗆 | 🗆 | 🗆 | 🗆 | 🗆 |
| An infection with chikungunya costs the patient a lot of money………………………. | 🗆 | 🗆 | 🗆 | 🗆 | 🗆 |
| Dengue is a serious disease……………………………………………………………………………… | 🗆 | 🗆 | 🗆 | 🗆 | 🗆 |
| People who get dengue can die of it……………………………………………………………….. | 🗆 | 🗆 | 🗆 | 🗆 | 🗆 |
| People who have dengue are limited in their social activities…………………………… | 🗆 | 🗆 | 🗆 | 🗆 | 🗆 |
| An infection with dengue leads to loss of income…………………………………………….. | 🗆 | 🗆 | 🗆 | 🗆 | 🗆 |
| An infection with dengue costs the patient a lot of money………………………………. | 🗆 | 🗆 | 🗆 | 🗆 | 🗆 |

| **Perceived benefits** | | | | | | |
| --- | --- | --- | --- | --- | --- | --- |
| If, during the coming rainy season, I check for mosquito breeding sites and eliminate them from my house and yard if necessary, … | | | | | | |
|  | 1 | 2 | 3 | 4 | 5 |  |
| I will not get chikungunya | 🗆 | 🗆 | 🗆 | 🗆 | 🗆 | I will get chikungunya |
| My family will not get chikungunya | 🗆 | 🗆 | 🗆 | 🗆 | 🗆 | my family will get chikungunya |
| My neighbours will not get chikungunya | 🗆 | 🗆 | 🗆 | 🗆 | 🗆 | my neighbours will get chikungunya |
|  |  |  |  |  |  |  |
| I will not get dengue | 🗆 | 🗆 | 🗆 | 🗆 | 🗆 | I will get dengue |
| My family will not get dengue | 🗆 | 🗆 | 🗆 | 🗆 | 🗆 | my family will get dengue |
| My neighbours will not get dengue | 🗆 | 🗆 | 🗆 | 🗆 | 🗆 | My neighbours will get dengue |

Fully agree

Do not agree at all

| **Perceived barriers** |  | | | | |
| --- | --- | --- | --- | --- | --- |
| During the coming rainy season I will **not** check for mosquito breeding sites and eliminate them if necessary because… |  |  |  |  |  |
|  | **1** | **2** | **3** | **4** | **5** |
| I do not know how to check for breeding sites……………………………………………………. | 🗆 | 🗆 | 🗆 | 🗆 | 🗆 |
| I do not like to eliminate breeding sites……………………………………………………….…….. | 🗆 | 🗆 | 🗆 | 🗆 | 🗆 |
| Neighbours do not do it either, so it makes no sense for me to do it……………….… | 🗆 | 🗆 | 🗆 | 🗆 | 🗆 |
| I never have mosquitoes in my house or yard……………………………………………………… | 🗆 | 🗆 | 🗆 | 🗆 | 🗆 |
| There are many other breeding sites the government does nothing about…………. | 🗆 | 🗆 | 🗆 | 🗆 | 🗆 |
| I cannot get rid of my garbage/debris/rubbish at another place than in my yard… | 🗆 | 🗆 | 🗆 | 🗆 | 🗆 |
| I live near a mondi/dam where more mosquitoes come from than from my yard. | 🗆 | 🗆 | 🗆 | 🗆 | 🗆 |
| Someone else from my family will do it already…………………………………………………… | 🗆 | 🗆 | 🗆 | 🗆 | 🗆 |
| I do not have the physical ability to check my yard every week………………………….. | 🗆 | 🗆 | 🗆 | 🗆 | 🗆 |
| I’m only at home when it is dark and I cannot see the breeding sites then…………. | 🗆 | 🗆 | 🗆 | 🗆 | 🗆 |

| **Self-efficacy** | | | | | | |
| --- | --- | --- | --- | --- | --- | --- |
| Checking my house and yard for mosquito breeding sites every week and if necessary eliminating them during the coming rainy season…. | | | | | | |
|  | 1 | 2 | 3 | 4 | 5 |  |
| is absolutely impossible for me | 🗆 | 🗆 | 🗆 | 🗆 | 🗆 | is absolutely possible for me |
| Is not manageable for me, even if I want to | 🗆 | 🗆 | 🗆 | 🗆 | 🗆 | is doable for me, if I want to |

| **Attitudes towards behaviour** | | | | | | |
| --- | --- | --- | --- | --- | --- | --- |
| Checking for mosquito breeding sites and if necessary eliminating them from my house and yard during the coming rainy season, for me is… | | | | | | |
|  | 1 | 2 | 3 | 4 | 5 |  |
| Unnecessary | 🗆 | 🗆 | 🗆 | 🗆 | 🗆 | Necessary |
| Tedious | 🗆 | 🗆 | 🗆 | 🗆 | 🗆 | Fun |
| Bad | 🗆 | 🗆 | 🗆 | 🗆 | 🗆 | Good |
| Stressful | 🗆 | 🗆 | 🗆 | 🗆 | 🗆 | Relaxing |
| Harmful | 🗆 | 🗆 | 🗆 | 🗆 | 🗆 | Beneficial |
| Worthless | 🗆 | 🗆 | 🗆 | 🗆 | 🗆 | Valuable |

| Whether I will check for mosquito breeding sites and if necessary eliminate them from my house and yard during the coming rainy season, …. | | | | | | |
| --- | --- | --- | --- | --- | --- | --- |
|  | 1 | 2 | 3 | 4 | 5 |  |
| is **not** dependent on what other people think of it | 🗆 | 🗆 | 🗆 | 🗆 | 🗆 | Depends on what other people think of it |

| **Subjective norms** | | | | | | |
| --- | --- | --- | --- | --- | --- | --- |
| My weekly checking for mosquito breeding sites and eliminating them from my house and yard during the coming rainy season will be …. | | | | | | |
|  | 1 | 2 | 3 | 4 | 5 |  |
| Disapproved of by people who are important to me | 🗆 | 🗆 | 🗆 | 🗆 | 🗆 | Approved by people who are important to me |
| Advised against by people whose opinion I value | 🗆 | 🗆 | 🗆 | 🗆 | 🗆 | Advised by people whose opinion I value |
| Discouraged by my neighbours | 🗆 | 🗆 | 🗆 | 🗆 | 🗆 | Encouraged by my neighbours |
| Not be expected from me | 🗆 | 🗆 | 🗆 | 🗆 | 🗆 | Expected from me |
| Not be stimulated by the government | 🗆 | 🗆 | 🗆 | 🗆 | 🗆 | Stimulated by the government |

| Checking house and yard for mosquito breeding sites every week and if necessary eliminating them during the coming rainy season, …. | | | | | | |
| --- | --- | --- | --- | --- | --- | --- |
|  | 1 | 2 | 3 | 4 | 5 |  |
| Is **never** done by people whose opinion is important to me | 🗆 | 🗆 | 🗆 | 🗆 | 🗆 | is **always** done by people whose opinion is important to me |
| Is **never** done by people who are important to me | 🗆 | 🗆 | 🗆 | 🗆 | 🗆 | is **always** done by people who are important to me |
| Is **never** done by my neighbours | 🗆 | 🗆 | 🗆 | 🗆 | 🗆 | is **always** done by my neighbours |

| **Perceived behavioural control** (recode: 1=5; 2=4; 3=3; 4=2; 5=1) | | | | | | |
| --- | --- | --- | --- | --- | --- | --- |
| Whether I will check for mosquito breeding sites and if necessary eliminate them from my house and yard during the coming rainy season, …. | | | | | | |
|  | 1 | 2 | 3 | 4 | 5 |  |
| is **not** dependent on help from other people | 🗆 | 🗆 | 🗆 | 🗆 | 🗆 | is dependent on help from other people |

| **Satisfaction on governmental mosquito breedingsite control** | | | | | | |
| --- | --- | --- | --- | --- | --- | --- |
| The government does enough to prevent and eliminate breeding sites | | | | | | |
|  | 1 | 2 | 3 | 4 | 5 |  |
| Do not **agree** at all | 🗆 | 🗆 | 🗆 | 🗆 | 🗆 | Fully agree |
